# Exercise Training-Induced Changes in MicroRNAs: Beneficial Regulatory Effects in Hypertension, Type 2 Diabetes, and Obesity

**DOI:** 10.3390/ijms19113608

**Published:** 2018-11-15

**Authors:** Alex Cleber Improta Caria, Carolina Kymie Vasques Nonaka, Ciro Silveira Pereira, Milena Botelho Pereira Soares, Simone Garcia Macambira, Bruno Solano de Freitas Souza

**Affiliations:** 1 Multicentric Program of Post-Graduate in Biochemistry and Molecular Biology, Federal University of Bahia, Bahia 40231-300, Brazil; alexcaria.personal@hotmail.com; 2 Center for Biotechnology and Cell Therapy, São Rafael Hospital, Bahia 41253-190, Brazil; carolina.nonaka@hsr.com.br (C.K.V.N.); ciro_bm@yahoo.com.br (C.S.P.); milena@bahia.fiocruz.br (M.B.P.S.); 3 Gonçalo Moniz Institute, Oswaldo Cruz Foundation (FIOCRUZ), Bahia 40296-710, Brazil; 4 National Institute of Science and Technology for Regenerative Medicine, Rio de Janeiro 21941-590, Brazil; 5 Department of Biochemistry and Biophysics, Health Sciences Institute, Federal University of Bahia, Bahia 40231-300, Brazil

**Keywords:** microRNAs, systemic arterial hypertension, type 2 diabetes mellitus, obesity, exercise training, microRNA

## Abstract

MicroRNAs are small non-coding RNAs that regulate gene expression post-transcriptionally. They are involved in the regulation of physiological processes, such as adaptation to physical exercise, and also in disease settings, such as systemic arterial hypertension (SAH), type 2 diabetes mellitus (T2D), and obesity. In SAH, microRNAs play a significant role in the regulation of key signaling pathways that lead to the hyperactivation of the renin-angiotensin-aldosterone system, endothelial dysfunction, inflammation, proliferation, and phenotypic change in smooth muscle cells, and the hyperactivation of the sympathetic nervous system. MicroRNAs are also involved in the regulation of insulin signaling and blood glucose levels in T2D, and participate in lipid metabolism, adipogenesis, and adipocyte differentiation in obesity, with specific microRNA signatures involved in the pathogenesis of each disease. Many studies report the benefits promoted by exercise training in cardiovascular diseases by reducing blood pressure, glucose levels, and improving insulin signaling and lipid metabolism. The molecular mechanisms involved, however, remain poorly understood, especially regarding the participation of microRNAs in these processes. This review aimed to highlight microRNAs already known to be associated with SAH, T2D, and obesity, as well as their possible regulation by exercise training.

## 1. Introduction

### 1.1. MicroRNAs

MicroRNAs (miRNAs) are small non-coding single-stranded RNAs, with approximately 22 nucleotides, that act in mechanisms of post-transcriptional regulation of gene expression [1]. miRNAs are abundant in prokaryotic and eukaryotic organisms, being widely distributed among species and frequently conserved [2]. The database miRBase 22 has cataloged 38,589 hairpin precursor miRNAs, which are associated with the expression of 48,885 mature miRNA products in 271 species [3,4]. The mechanisms of action of miRNAs rely on the recognition and binding to the 3’-untranslated region (3’-UTR) of the target mRNAs [5]. This can lead to mRNA degradation, deadenylation, or the inhibition of translation [6,7].

It is estimated that miRNAs are involved in the regulation of up to 60% of the protein-coding genes in the human genome [8,9,10]. Furthermore, the individual genetic background can affect the ability of miRNAs to bind to target mRNA and regulate gene expression. Approximately 11% of the currently known single nucleotide polymorphisms (SNPs) are located in the 3’-UTR of several genes, which may interfere with the miRNA-mRNA interaction due to the destruction of the binding site or through the creation of new binding sites [11,12]. A significant number of SNPs are also located within the sequences of pri-, pre-, and mature miRNAs [11]. There is growing evidence of the involvement of such gene polymorphisms in the development of a great number of diseases such as hypertension, diabetes, obesity, and cardiovascular diseases [13,14].

Besides the role of individual variability, one of the many challenges involved in the study of miRNAs’ regulatory network is the ability of each single miRNA to regulate multiple targets, which adds complexity to the analysis and interpretation of the results [15]. It can be challenging to establish the biological significance of altered microRNA profiles in different settings, tissues, and compartments [16,17]. Nevertheless, it is clear that miRNAs promote a fine-tune regulation of a variety of biological processes that include cell metabolism, maturation, survival, proliferation, differentiation, and apoptosis. Therefore, miRNAs can have their expression altered under physiological conditions, and may be involved in the pathogenesis of diseases [18,19].

### 1.2. MiRNAs as Mediators and Biomarkers of Cardiovascular Diseases

MiRNAs are promising tools for the diagnosis, prognosis, or therapeutic guidance, applied as biomarkers of cardiovascular diseases. The discovery of altered miRNA expression profiles, defining a molecular signature for specific diseases, may increase pathophysiological knowledge and help in identifying novel therapeutic targets. Circulating miRNAs are remarkably stable, being transported as (i) ribonucleoprotein complexes, principally Argonaute, (ii) associated with high- and low-density lipoproteins or (iii) inside extracellular vesicles [20,21,22]. Studies on extracellular vesicles (EVs) date back to the 1960s and, since then, the role of EVs to cell-to-cell communications have been well established. EV’s allow for the stable transport of microRNAs, protecting them from enzymatic and physical degradation, allowing for their assessments and applications as biomarkers and/or therapeutic targets [23,24,25]. Both intracellular and circulating miRNAs can be found dysregulated in cardiovascular diseases, which facilitates their application as novel biomarkers [20,26].

Cardiovascular diseases are estimated to affect 422.7 million people and are the largest contributors to worldwide mortality [27]. The death rate associated with systemic arterial hypertension (SAH) increased by 34.7% in the latest years [28]. Cardiovascular events are the main cause of death in patients with diabetes mellitus [29]. Type 2 Diabetes mellitus (T2D) is a highly prevalent metabolic disease, with more than 400 million currently affected people in the world. It is estimated that more than 640 million will be affected by T2D by 2040 [30]. Diabetes can progressively lead to cardiac dysfunction, representing an independent risk factor for cardiac diseases [31]. In 2013, the American Medical Association recognized obesity as a disease, which is now considered to be a worldwide epidemic [32]. This is a progressive and multifactorial disease characterized by an increase of white adipose tissue due to both hypertrophy and hyperplasia of adipocytes [33]. Moreover, obesity is highly associated with T2D and cardiovascular diseases [34,35,36]. The involvement of dysregulated miRNAs in SAH, T2D, and obesity is reviewed below.

## 2. MiRNAs and Systemic Arterial Hypertension (SAH)

SAH is clinically defined as a persistent non-physiological elevation of systemic arterial pressure. It is considered to be a multifactorial clinical condition, which develops as the result of genetic predisposition and environmental factors [37]. Hypertension is one of the main etiologies of cardiovascular diseases, strokes, and kidney failure [38], leading to premature death and disability. It is associated with 45% of deaths due to heart disease and 51% of deaths due to strokes [39]. SAH is an independent risk factor for ischemic cardiomyopathy and is present in 64% of patients with acute myocardial infarction. There are several risk factors for SAH, which can be classified as non-modifiable risk factors—age, ethnicity, gender, and genetics—or modifiable risk factors, such as sedentary lifestyle, psychological stress, smoking, obesity, alcoholism, caffeine consumption, diabetes mellitus type 2, and others [40].

**Figure 1:**
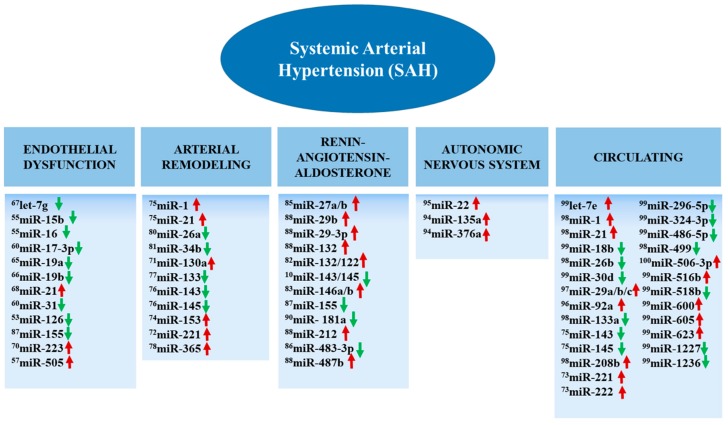
The dysregulated miRNA profile in Hypertension. The diagram shows the miRNA expression pattern associated with different pathophysiological mechanisms involved in hypertension, including endothelial dysfunction, arterial remodeling, renin-angiotensin-aldosterone system (RAAS), and autonomic nervous system. Circulating miRNAs evaluated in serum, plasma, whole-blood, or mononuclear cells, in clinical studies, are also listed. ↑ = upregulated, ↓ = downregulated.

Different pathophysiological processes involved in SAH are now known to be regulated by miRNAs, including endothelial dysfunction, dysregulation of vascular smooth muscle cells (VSMCs), the increase of sympathetic system activity, and alterations in renin-angiotensin-aldosterone system (RAAS) [41,42]. The results of several experimental studies, both in vitro and in vivo, as well as clinical studies, revealed that the expression of different miRNAs can be altered in the context of SAH (Figure 1).

### 2.1. MiRNAs and Endothelial Dysfunction in SAH

Normal endothelial functions include the maintenance of vascular integrity and homeostasis, leukocyte adhesion, the regulation of vascular tone, and tissue repair [43,44]. In SAH, however, endothelial dysfunction occurs in response to a high shear stress in the vessel wall, which is associated with modifications of intracellular pathways and the impaired expression of constitutive endothelial nitric oxide synthase (eNOS) [45]. Several miRNAs are involved in the link between endothelial dysfunction and hypertension by controlling endothelial cell gene expression and function in the physiological and pathological states [46].

It is known that the vascular endothelial growth factor (VEGF) signaling is altered in SAH. Under physiological conditions, VEGF is not only involved in angiogenesis but also in blood pressure regulation. In fact, the use of anti-VEGF therapies can be associated with the development of SAH as an adverse effect [47]. In the last decade, a considerable amount of data regarding the regulation of VEGF signaling by miRNAs has emerged [48,49].

MiR-126 is highly expressed in the cardiac endothelium, being involved in the regulation of the VEGF signaling pathway. MiR-126 participates in processes of angiogenesis and the maintenance of vascular integrity, by targeting the PI3K regulatory subunit p85 β (PIK3R2), which leads to modulation of the PI3K/Akt signaling pathway in endothelial progenitor cells [50]. In addition to PIK3R2, miR-126 also inhibits SPRED-1 (Sprouty-related, EVH1 domain-containing protein 1), another negative regulator of angiogenesis that acts by inhibiting VEGF signaling pathway. Indeed, when normally translated, PI3KR2 and SPRED-1 inhibit RAF1 and PI3K, which are required for the activation of the VEGF pathway. Therefore, inhibition of PI3KR2 and SPRED-1 allows for the activation of the VEGF pathway and, consequently, favors angiogenesis under physiological conditions [51]. Transgenic mice lacking the miR-126 expression present developmental abnormalities that include vascular leakage, hemorrhaging, and partial embryonic lethality [52]. These findings were attributed to the compromised signaling of proangiogenic growth factors in the absence of miR-126.

The MiR-126 expression was reported to be reduced in peripheral blood mononuclear cells (PBMCs) of hypertensive patients and, along with miR-9, showed a negative correlation with the 24-h mean pulse pressure [53]. Decreased levels of miR-126 in endothelial cells favor inflammation, as demonstrated by the increased Tumor necrosis factor alpha (TNF-α)-stimulated expression of vascular cell adhesion molecules (VCAM-1) in endothelial cells transfected with a miR-126 inhibitor [54]. MiR-15b and miR-16 are also involved in the regulation of angiogenesis and vascular function. Both miRNAs have VEGF mRNA as a direct target and were found to be downregulated in hypoxic conditions [55].

In addition to VEGF, fibroblast growth factors (FGFs) are also strong promoters of angiogenesis [56]. FGF18, a proangiogenic factor, is repressed by miR-505 in endothelial cells and was found up-regulated in the plasma of hypertensive patients [57]. Interestingly, miR-505 also presented a non-significant trend to be increased in pre-hypertensives subjects, when compared to controls, also suggesting a potential role as a biomarker of pre-hypertension.

MiR-17-3p and miR-31, shown to be altered in SAH, promote vascular inflammation through the modulation of the expression of VCAM-1, ICAM-1, and E-SEL [58,59,60]. Moreover, vascular inflammation is associated with increased oxidative stress, which can lead to eNOS uncoupling, decreased nitric oxide (NO) production (contributing to endothelial dysfunction) and decreased vasodilation ability [61,62]. MiR-155 regulates endothelium-dependent vasodilation by reducing the eNOS mRNA [63] and also plays a role as an anti-angiogenic miRNA in the regulation of adaptive neovascularization [64]. Additionally, miR-19a presents anti-proliferative properties in endothelial cells by inhibiting cyclin D1 mRNA [65], while miR-19b reduces the apoptosis of endothelial cells exposed to TNF-α in vitro [66]. Let-7g, miR-21, and miR-223 may also play a role in apoptosis of endothelial cells [67,68,69]. Interestingly, the level of miR-223 was shown to be increased during the establishment and progression of hypertension-induced heart failure in an experimental model in rats [70].

### 2.2. MiRNAs and Arterial Remodeling in SAH

The proliferation of VSMCs is one of the hallmarks of the vascular response in SAH, a process leading to the structural remodeling of the arteries, with lumen reduction [71]. Vessel wall damage induces changes in gene expression profile and phenotype of VSMCs, increasing their proliferation, migration, and collagen synthesis, while the expression of contractile proteins is decreased [9]. Several miRNAs have already been reported to participate in the regulation of the VSMCs’ phenotype, including miR-221, -222, -153, -143, -145, -133, -21, -1, -130a, -365, and -26a, which are discussed below.

MiR-221 regulates the phenotype of VSMCs by reducing their contractile profile in response to the platelet-derived growth factor (PDGF) [72]. Interestingly, circulating levels of miR-221 and miR-222 were found to be increased in hypertensive patients when compared to the controls and were even higher in hypertensive patients that presented left ventricular hypertrophy, which suggests a possible role of these two microRNAs in the complications associated with SAH [73]. MiR-153 was found increased in VSMCs in spontaneously hypertensive rats and may compromise the contractile phenotype by targeting the Potassium Voltage-Gated Channel Subfamily Q Member 4. This channel controls arterial contraction and is compromised in hypertension due to miR-153 overexpression [74].

In hypertensive patients, reduced circulating levels of miR-143, miR-145, miR-133 and the increased expression of miR-21 and miR-1 were found [75]. The MiR-143/145 cluster is highly expressed in VSMCs under physiological conditions and plays a role in the differentiation of stem/progenitor cells into VSMCs. The underexpression of the miR-143/145 cluster in SAH may influence the contractile phenotype of VSMCs [10,75,76]. MiR-133 is a negative regulator of VSMCs’ proliferation [77], while miR-21 upregulation was shown to be involved in the processes of proliferation and survival of VSMCs [19].

Other miRNAs were shown to be involved in the regulation of the proliferative response of VSMCs. MiR-130a inhibits the growth arrest-specific homeobox, contributing to the VSMCs’ proliferation [71]. In contrast, miR-365 inhibits the proliferation of VSMCs by down-regulating cyclin D1 expression. Various stimuli, including angiotensin II signaling, lead to the downregulation of miR-365, which, in turn, results in an increased VSMC proliferation [78,79]. MiR-26a promotes aberrant VSMC proliferation, being involved in the regulation of SMAD-1 and SMAD-4, two members of the TGF-β (Transforming Growth Factor β) signaling pathway [80]. In spontaneously hypertensive rats, the downregulation of miR-34b was observed, which favored the proliferation of VSMCs by increasing the levels of cyclin-dependent kinase 6 (CDK6) [81].

### 2.3. MiRNAs and Renin-Angiotensin-Aldosterone System (RAAS) in SAH

MiRNAs participate in RAAS-mediated cardiovascular inflammation. Some miRNAs have been linked to the RAAS signaling, such as miR-155, miR-146a/b, miR-132/122 cluster, and miR-483-3p [82,83,84]. MiR-145, miR-27a/b, and miR-483-3p inhibit ACE expression, an enzyme that plays a crucial role in the regulation of blood pressure [85,86]. Interestingly, the miR-143/145 cluster is underexpressed in SAH, correlating with a higher expression of ACE [10].

Different miRNAs that target angiotensin II mRNA, including miR-483-3p and miR-155, are downregulated in SAH, leading to an increased angiotensin II expression [86,87]. The persistently increased production of angiotensin II facilitates the development of cardiac hypertrophy in SAH through actions of the following miRs: miR-487b, miR-29b, miR-29-3p, miR-212, and miR-132 [88].

Finally, it was demonstrated that miR-181a inhibits renin mRNA in a genetically hypertensive mouse strain [89] Additionally, MiR-181a has been shown to bind to the 3’-UTR of renin mRNA [90] and was found downregulated in hypertensive mice, correlating with an increased expression of renin and angiotensin II [89]. In hypertensive human subjects, however, a different result was observed, since miR-181a expression was increased in the serum and positively correlated with systolic and diastolic blood pressure, independently of renin levels [91].

### 2.4. MiRNAs and Autonomic Nervous System in SAH

Neurogenic abnormalities in specific sites of central blood pressure control also lead to hypertension. Central neuronal mechanisms of blood pressure involve particular anatomic structures in the brainstem, such as the nucleus of the solitary tract and rostral ventrolateral medulla which play different roles in pressure control. Elevated sympathetic nervous system activity contributes to the overactivation of the RAAS, vascular injuries, remodeling, and endothelial dysfunction. Vascular damage due to inflammation [92] and angiotensin signaling alteration [93] is the main mechanism that leads to neurogenic hypertension. In spontaneously hypertensive rats, this is associated with the dysregulation of miR-135a and miR-376a [94].

Chromogranin A (Chga) is a molecule involved in the central and peripheral blood pressure controls and also in the pathogenesis of SAH, since neuronal Chga-granules store catecholamines in the brainstem and release these neurotransmitters to regulate sympathetic outflow to the periphery. In spontaneously hypertensive rats, the adrenal glands and plasma are elevated but its central expression is decreased, thus, miR-22 was associated with the dysregulation of Chga in brainstem cardiovascular control nuclei, contributing to the pathogenesis of SAH in spontaneously hypertensive rats [95].

These results indicate the contribution of miRNAs to the regulation of blood pressure in physiological and pathophysiological conditions by different peripheral and neuronal cells, which reveals the potential of miRNAs as important biomarkers for the diagnosis of SAH, and, future, as therapeutic targets.

### 2.5. Circulating miRNAs in SAH

In addition to the miRNAs described above, a number of studies have focused on the search for circulating miRNAs that could serve as SAH biomarkers in the future. As mentioned before, circulating miRNAs have the potential to become novel biomarkers, since they are involved in different biological processes, may be detected during early disease pathogenesis, are easily extracted from peripheral blood, and remarkably stable.

Different studies point to different profiles of miRNA expression in the circulation. Plasma levels of miR-92a were found to be increased in hypertensive subjects and to correlate with 24 h mean systolic and diastolic pressure [96]. In another study, plasma levels of mir-29a, b, and c were increased in subjects with hypertension, with positive correlations with office systolic and diastolic blood pressure, office pulse pressure, 24 h mean systolic and diastolic blood pressure, and 24 h mean pulse pressure [97]. The increased expression of miR-1, miR-21, miR-208b, and miR-499, accompanied by decreased levels of miR-133a and miR-26b, were found in PBMCs of hypertensive subjects, being associated with left ventricular hypertrophy [98]. Plasma levels of miR-516b, miR-600, miR-605, miR-623, and let-7e were increased in hypertensive subjects, while miR-18b, miR-30d, miR-296-5p, miR-324-3p, miR-486-5p, miR-518b, miR-1236, and miR-1227 were decreased when compared to the controls [99]. A recent study revealed that miR-506-3p is elevated in the peripheral blood of hypertensive subjects, also showing a correlation with the stage of hypertension [100]. The lack of consensus in these studies may be influenced by factors such as sample diversity, the degree of disease severity, comorbidities, therapeutic approach, and disease staging.

### 2.6. Single Nucleotide Polymorphisms and miRNAs in SAH

SNPs in the 3’-UTR of diverse genes are involved in the establishment of a hypertensive phenotype. Genetic predisposition to essential hypertension was associated with the ss52051869 SNP, which is located in the 3’-UTR of *SLC7A1*, the L-arginine transporter gene, and leads to endothelial dysfunction and disturbances in the L-arginine and nitric oxide metabolism [101]. Later, it was demonstrated that this SNP creates miRNA-122 binding sites, decreasing *SLC7A1* expression in hypertensive patients. The rs5068 (A/G) allele in the 3’-UTR of the *NPPA* gene, that encodes atrial natriuretic peptide (ANP), is also involved in hypertension. It was shown that miR-425, a specific microRNA expressed in the human atria and ventricles, binds to the sequence spanning rs5068 in an allele-specific manner at the A allele, conferring a regulatory role for microRNA-425 in ANP expression [102]. In 2014, Maharjan and colleagues demonstrated that an SNP located in the *Cyp11B2* gene, which encodes for human aldosterone synthase, creates a binding site for miR-766. The binding of miR-766 to the 735G-allele of *Cyp11B2* leads to a reduction in the expression of human aldosterone synthase and decreased blood pressure levels [103]. An SNP at the 3’-UTR of the *ATF1* (activating transcription factor 1) gene altered the posttranscriptional regulation of this gene by miRNAs, leading to essential hypertension with high ATF-1 expression [104]. The ATF1 rs11169571 allele is involved in the pathogenic mechanism of essential hypertension by altering the hsa-miR-1283 binding site. Another SNP associated with essential hypertension is located at the 3’-UTR of the *NET* gene, which encodes the norepinephrine transporter (NET), responsible for the removal of Norepinephrine from the neuroeffector junction. The presence of the T allele of rs7194256 (C/T) is strongly associated to cardiovascular abnormalities such as an elevated larger left ventricular mass index, systolic and diastolic blood pressures, augmentation, and uncontrolled heart rate. The rs7194256 SNP in the 3’-UTR of the *NET* gene at T allele has a binding site for the microRNA miR-19a-3p. This miRNA is significantly reduced in patients that have clinical symptoms of NET impairment due to high levels of Norepinephrine circulating, which lowered the miR-19a-3p levels.

## 3. MiRNAs and Type 2 Diabetes Mellitus

T2D continues to grow in terms of worldwide prevalence, being associated with a high morbidity and mortality [30]. T2D develops as the result of combined genetic predisposition and environmental factors, which include a hypercaloric diet, obesity, and a sedentary lifestyle [105]. Insulin resistance in target tissues is the primary pathogenic mechanism of T2D that leads to hyperglycemia. A constant glucose elevated level is a permanent insult to the expansion of β-cell mass to increase insulin secretion. However, this compensatory mechanism to maintain glucose homeostasis fails in T2D patients, leading to chronic hyperglycemia and hyperinsulinemia [106]. The molecular mechanisms underlying insulin resistance, impairment of insulin signaling, and exhaustion of pancreatic β-cells are not completely elucidated. 

**Figure 2:**
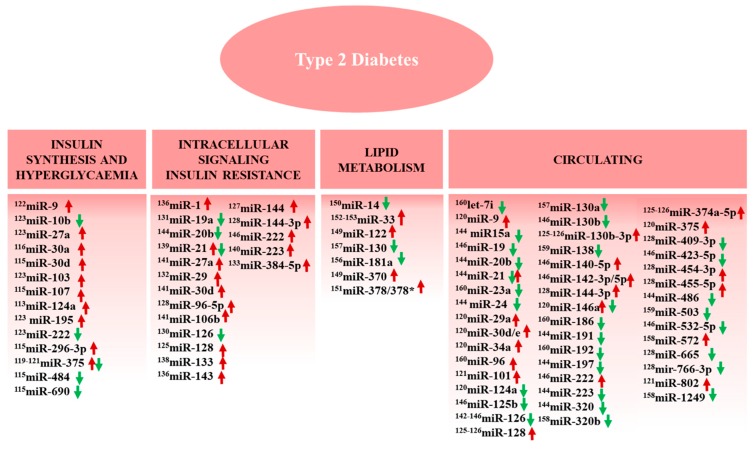
The dysregulated miRNA profile in Type 2 Diabetes. The diagram shows the miRNA expression pattern associated with different pathophysiological mechanisms involved in type 2 Diabetes, including insulin synthesis, intracellular signaling/insulin resistance, lipid metabolism, and hyperglycemia. Circulating miRNAs evaluated in serum, plasma, whole-blood, or mononuclear cells, in clinical studies, are also listed. ↑ = upregulated, ↓ = downregulated, ↑↓ = studies differ and report either increased or decreased expression of the miRNA.

The evaluation of circulating miRNAs, including those transported by extracellular vesicles (EVs), could help to reach an early diagnosis of T2D, risk stratification, monitorization of disease progression, and therapeutic guidance. The EVs participate in intercellular communication interfering in many biological processes such as cell proliferation and differentiation, immunomodulation, homeostasis, and neurological signaling. EVs are also involved in the pathogenesis of diseases, including T2D [107,108,109]. The profile of microRNAs transported inside EVs can be associated with aging, changes in tissue microRNA signatures, receptor signaling dysregulation, and gene expression alterations [110,111]. The regulation of microRNA expression is associated with different comorbidities and complications of T2D, including impaired angiogenesis and micro and macrovascular injury [108]. Several miRNAs known to be altered in T2D (Figure 2) are described below.

### 3.1. MiRNAs, Insulin Synthesis, and Secretion

High blood glucose levels lead to increased insulin production by increasing transcription, mRNA stability and translation in pancreatic β cells [112]. Different miRNAs, including miR-124a [113,114], miR-107 [115], miR-30a [116], and miR-30d [115] are involved in the regulation of insulin transcription and translation in response to high glucose. The downregulation of miR-484, miR-690, and miR-296 was observed in diabetic mouse pancreatic islets, being associated with the inhibition of insulin transcription [115]. Interestingly, the upregulation of miR-296-3p was associated with pancreatic cell resistance to apoptosis in a pro-inflammatory environment [117]. Besides regulating gene expression by translational repression, miRNAs can also be tissue-specific or exhibit a developmental-stage-specific expression, which could modulate different biological processes. One of these processes is insulin secretion, being regulated by different miRNAs, including miR-375 [112], and miR-9 [118].

The molecular mechanisms involved in the chronic adaptation of pancreatic β-cells to hyperglycemia are not fully elucidated. Different experimental models were used to investigate the role of different miRNAs in the control of intracellular pathways critical to insulin signaling, such as PI3K-PDK1-AKT (phosphatidylinositol-4,5-bisphosphate 3-kinase/Phosphoinositide-dependent protein kinase-1/AKT/Protein kinase B (PKB), serine/threonine-specific protein kinase). An in vitro study demonstrated that miR-375 reduces PDK1 levels, impairing the glucose-mediated increase in insulin expression. Moreover, miR-375 was found downregulated in pancreatic islets from Goto-Kakizaki (GK) type 2 diabetic rats [119]. In contrast, miR-375 was found upregulated in the serum of newly diagnosed T2D patients [120]. Indeed, another study reported increased serum levels of miR-375 in T2D patients, along with miR-101 and miR-802 [121].

Insulin secretion can also be affected by the overexpression of miR-9, which targets the transcription factor Onecut-2, leading to the increased expression of Granuphilin/Slp4, a negative regulator of insulin secretion [122]. Moreover, it has been demonstrated that miR-222, miR-27a, miR-195, miR-103, and miR-10b expression patterns varied with hyperglycemia in insulin-target tissues of non-obese diabetic rats, suggesting a role in the disease pathogenesis [123].

### 3.2. MiRNAs and Insulin Resistance

A high-fat and sugar diet is associated with hyperglycemia, hyperlipidemia, and insulin resistance. In this context, the pattern of phosphorylation in serine residues of insulin receptor substrate-1 (IRS-1) and insulin receptor substrate-2 (IRS-2) can be altered. In healthy individuals, IRS-1 and IRS-2 tyrosine residues are usually phosphorylated, leading to the activation of insulin signaling cascades [124]. When the phosphorylation of serine residues occurs, however, reduced stimulation of these receptors by phosphoinositide 3-kinase (PI3K) occurs, leading to the reduced activation of the PI3K/AKT/mTOR (mammalian target of rapamycin) pathway and impaired translocation of GLUT (glucose transporter)-4-containing vesicles to the cell membrane, decreasing glucose uptake [31].

The expression of IRS-1, a key protein in the insulin cascade, is negatively regulated by different miRNAs, including miR-128, which was found to be increased in the serum of pre-diabetic and T2D subjects, along with miR-130b-3p and miR-374a-5p [125,126]. IRS-1 levels also showed a negative correlation with increased miR-144 expression in the whole blood of T2D subjects, exhibiting a correlation with progressively increased glycemic status [127]. In another study, miR-96-5p, which also targets IRS-1, was found overexpressed in the serum of T2D subjects, along with miR-144-3p, miR-454-3p, and miR-455-5p, while miR-409-3p, miR-665, and miR-766-3p were reduced [128]. IRS-1 is also negatively regulated by miR-126, which was found to be overexpressed by hepatocytes in an in vitro model of insulin resistance [129]. MiR-126 was also found to be over-expressed in the adipose tissue of hyperinsulinemic obese mice, targeting IRS-1, and, consequently, impairing glucose uptake [130].

The translocation of GLUT to the plasma membrane of target cells must occur in order to promote glucose uptake. Specific miRNAs interfere in the insulin signaling pathway by targeting the PI3K/AKT axis, which is involved in the translocation of GLUT4 to the cell surface. This is the case for miR-19a [131], miR-29 [132], and miR-384-5p [133], with altered expression in T2D. MiR-29 inhibits the insulin-stimulated glucose uptake, and promotes insulin resistance, resulting in the development of T2D [134,135]. The mechanisms involved in this regulation consist in the reduction of AKT phosphorylation, mediated by miR-29, miR-1, and miR-33a/b, or in the direct regulation of AKT mRNA mediated by miR-143 [136]. Furthermore, GLUT4 transcription is regulated by the transcription factor Kruppel-like 15 (KLF15), which is targeted by the miR-133 family [137,138]. MiR-223 and miR-21 also participate in the regulation of GLUT4 translation, as demonstrated by in vitro studies [139,140]. In a model of T2D induced by high-fat diet/streptozotocin in rats, miR-106b, miR-27a, and miR-30d were upregulated, leading to the inhibition of GLUT4, MAPK 14, and PI3K [141].

Clinical studies have consistently shown that circulating levels of miR-126 are downregulated in T2D patients [142,143,144,145,146]. The MiR-126 expression was decreased before the manifestation of T2D, correlating with disease onset [143] and high blood glucose levels [144]. Plasma levels of miR-126, as well as miR-20b, miR-21, miR-24, miR-15a, miR-191, miR-197, miR-223, miR-320, and miR-486, were found to be reduced, while miR-28-3p was increased in T2D subjects [144]. In another study, miR-126 was also found to be reduced in the plasma of T2D subjects, along with miR-423-5p, miR-125b, miR-192, miR-130b, miR-19, and miR-532-5p, while miR-140-5p, miR-142-3p, and miR-222 were upregulated [146]. Interestingly, progressively lower circulating levels of miR-126-3p and miR-21-5p were found in healthy controls compared to T2D subjects and T2D with complications [145]. It is possible that the downregulation of miR-126 counteracts angiogenesis, since miR-126 was also found with a reduced expression in endothelial progenitor cells obtained from T2D patients, correlating with a decreased proliferation, migration, and increased induction of apoptosis, through the disinhibition of SPRED1, which in its turn inhibits the Ras/ERK/VEGF and PI3K/Akt/eNOS signal pathways [147]. These data suggest that miR-126 may be involved in the pathogenesis of micro- and macrovascular complications of T2D.

### 3.3. MiRNAs and Lipid Metabolism

It is well established that insulin resistance is associated with changes in the expression of miRNAs that regulate the lipid metabolism. Hepatic expression of miR-122 is involved in cholesterol and fatty acid metabolism [148]. The increased expression of miR-370 activates the expression of miR-122 and affects the lipid metabolism, increasing hepatic triglycerides accumulation [149]. MiR-14 also regulates the fat metabolism, as shown by the increased levels of triacylglycerol and diacylglycerol found in miR-14 null animals [150]. MiR-378/378* (MiR378-5p and 378-3p) play important roles in regulating lipid metabolism and their overexpression increases triacylglycerol during adipogenesis [151], while miR-33 targets genes involved in cholesterol transport (ABCA1) and controls HDL (High Density Lipoprotein) levels in vivo [152,153].

It has been reported that Sirtuin 1 (SIRT1) regulates the glucose and lipid metabolism [154]. MiR-181a regulates SIRT1, increasing the insulin sensitivity in hepatocytes [155]. The increased cardiac expression of p53 and p21 and the decreased expression of miR-181a was reported in diabetic subjects, suggesting a possible role for this microRNA in the development of diabetic cardiomyopathy [156]. Levels of miR-130a were found to be reduced in the peripheral blood of T2D subjects and in the omental adipose tissue of T2D subjects, while the target mRNA levels—PPARγ—were elevated [157].

### 3.4. Circulating miRNAs in T2D

The evaluation of expression levels of different miRNAs in plasma samples revealed that the pattern of expression of miR-1249, miR-320b, and miR-572 can distinguish T2D patients, prediabetes, and controls [158]. Moreover, lean diabetic patients can be distinguished from obese diabetic patients by the combined evaluation of miR-503 and miR-138 levels [159]. A prediction analysis of genes regulated by these miRNAs points to different biological functions, which include development, signal transduction, cell survival, cell differentiation, cell proliferation, apoptosis, cell metabolism, and ion transport regulation. In another study, decreased plasma levels of miR-15a, miR-20b, miR-21, miR-24, miR-126, miR-191, miR-197, miR-223, miR-320, and miR-486 were found in T2D subjects [144]. Although these studies identified many different miRNA that could help reach an early diagnosis and acting as novel therapeutic targets, their function in the pathogenesis of T2D is far from being clarified.

Interestingly, miR-126 was associated with an increased risk for development of diabetes in the future, but the correlation with the other miRNAs detected in T2D is not fully elucidated [144]. Kong and colleagues, 2011, investigated the role of seven miRNAs that had been previously described as key regulatory elements in insulin synthesis, secretion, and signaling, as well as free fatty acid action on pancreatic β-cells. They found increased levels of miR-9, miR-29a, miR-30d, miR-34a, miR-124a, miR-146a, and miR-375 in serum samples of newly diagnosed T2D patients, which differed from pre-diabetes individuals with impaired glucose tolerance and those without it [120]. So, the profile of miRNA expression varies with the stage of the disease. Yang and colleagues revealed low serum levels of miR-23a, let-7i, miR-486, miR-96, miR-186, miR-191, miR-192, and miR-146a in T2D subjects [160].

### 3.5. Single Nucleotide Polymorphisms and miRNAs in T2D

Different gene variants have been reported to increase the susceptibility or to participate in the pathogenesis of T2D [161,162]. SNPs in different genes involved in T2D pathogenesis were reported, including transcription factor 7-like 2-TCF7L2 [163], Angiotensin-converting enzyme [164], Adiponectin [165], IGF2 receptor [166], and Sirtuins—SIRT [167]. Insulin-like growth factor (IGF) and its receptor (IGF type 2 receptor: IGF2R) are involved in a variety of physiological events including glucose homeostasis [168]. Genetic variants of this receptor were associated with diabetes [169,170,171]. The evaluation of the IGF2R genotypes distributions in diabetes patients revealed that the CC allele had a higher frequency among these patients than the others investigated alleles (CT or TT), thus affecting the circulating IGF2R levels distribution and T2D development [166]. However, the role of genetic variants of the IGF2R system in type 2 diabetes mellitus is still unclear. Lv and colleagues (2008) demonstrated that the ACAA-insertion/deletion polymorphism in IGF2R 3’-UTR is located within some specific miRNAs binding sites that result in the individual variability of IGF2R expression [172]. It was demonstrated in a prediction analysis and in vitro experiments that among the miRNA identified the hsa-miR-657 was involved in the inhibitory interaction with 3’-UTR of the *IGF2R* gene, resulting in the modulation of IGF2R expression in an allele-specific manner.

The Lipoprotein lipase (LPL) is a key enzyme in lipid metabolism and abnormalities in its function are associated with dyslipidemia, insulin resistance, and T2DM. A study conducted in Iranian DM carriers analyzed the correlation between SNP rs13702 (C/T) in the 3’-UTR binding site to miRNA-410 of *LPL* gene and T2DM susceptibility. This SNP leads to an impairment in the LPL system due to dysfunction in gene expression negative regulation by miRNA-mRNA interaction. This abnormality has as one of its features the higher circulating levels of LPL that leads to ectopic fat deposition in peripheral tissues and contributes to insulin resistance, hyperglycemia, and progressively to T2DM [173].

Polymorphisms in the sequence of pri-miRNA have been reported for miR-124a, which regulates pancreatic islet development and insulin secretion by targeting Foxa2 and Rab27a [113,114]. Regarding the genetic variations in pre-miRNA, the overexpression of miR-34a was associated with cell apoptosis and the impairment of insulin secretion due to p53 activation [174]. Finally, the variations in regulatory regions affect the transcription initiation of miRNA encoding genes, altering the expression of miRNAs. Let-7 is involved with the regulation of the synthesis and secretion of insulin. Indeed, let-7 knockdown in high-fat mice was associated with improved insulin sensitivity in target tissues [175]. By the prediction analysis, it was demonstrated that a genetic variant polymorphism (rs1143770) in a regulatory region of miRNA let-7a-2 is associated with diabetic nephropathy. The proposed mechanism to explain the finding is based on the commitment of the transcription factor cyclic adenosine monophosphate response element-binding protein (CREB), which is associated with a susceptibility to diabetic nephropathy [176]. Insulin resistance is related to the diverse complications of T2D and chronic inflammation also contributes to altering the miRNA function and expression. MiR-129 was expressed at a lower level in impaired fasting glucose patients and T2DM, however, the specific polymorphism associated is still unknown [127].

Wang and colleagues (2015) evaluated the possible association of genetic polymorphisms in miR-27a, miR-146a, and miR-124a with T2D [177]. Although no significant correlations were detected between the selected genotypes and T2D development, the authors found correlations between a miR-27a SNP (rs895819) and a miR-124a SNP (rs531564) with T2D associated with being overweight, while a protective effect of rs531564 in miR-124a was described. Altogether, these findings reinforce the role of miRNA polymorphisms in T2D and the relevance of environmental factors in association with miRNA polymorphisms to determine the disease development.

## 4. MiRNAs and Obesity

**Figure 3:**
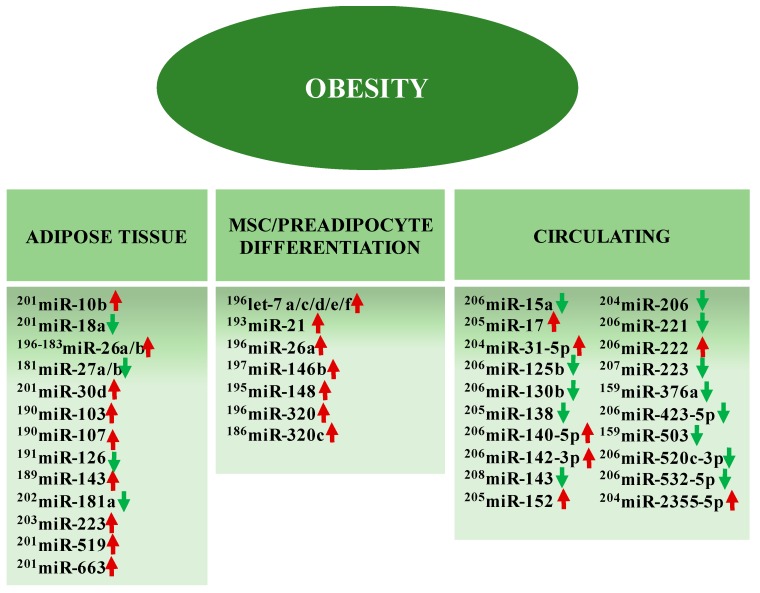
The dysregulated miRNA profile in obesity. The diagram shows the miRNA expression pattern found in the adipose tissue and during MSC/preadipocyte differentiation. Circulating miRNAs evaluated in serum, plasma, whole-blood, or mononuclear cells, in clinical studies, are also listed. ↑ = upregulated, ↓ = downregulated.

Obesity is considered an inflammatory disease that contributes to the development of SAH, T2D and coronary heart disease [178]. Different miRNAs participate in the adipogenesis process and modulate signaling pathways related to cell proliferation and differentiation, insulin sensitivity, and lipid metabolism [179]. Other miRNAs may be involved with inflammatory processes in obesity, either in the adipose tissue or systemically (Figure 3).

### 4.1. MiRNAs Involved with Adipocyte Differentiation and Proliferation

During the process of adipocyte differentiation and proliferation, the expression of miR-27a and 27b is reduced, while Peroxisome proliferator-activated receptor γ (PPARγ), one of their targets is, in turn, overexpressed [180,181]. PPARγ is a member of nuclear receptors that regulate the transcription of several genes associated with obesity and diabetes [182]. It stimulates adipocyte differentiation and proliferation in order to increase reserves of fat in the adipose tissue. It also promotes the activation of LPL in adipose tissue, which decreases the amount of free fatty acids (FFA) present in plasma lipoproteins by capturing these lipids and storing them in adipose tissue in the form of triacylglycerol. In this way, FFA decreases, while the sensitivity of the insulin receptor increases.

MiR-26a and miR-26b, along with other microRNAs, are involved in adipocyte differentiation and proliferation [181,183,184,185,186,187]. The increased expression of miR-26a and miR-26b leads to the downregulation of phosphatase and tensin homologue (PTEN), which inhibits the AKT/PKB signaling pathway, impairing GLUT translocation. The deletion, mutation, or reduction of PTEN has several clinical implications, including insulin sensitivity and obesity [188]. Other miRNAs, including miR-103, miR-107, and miR-143, regulate hepatic insulin sensitivity in obese individuals through different mechanisms [189,190].

MiR-126 was found to be downregulated in obesity and this may be involved in several processes since one of its targets in adipocytes is PI3KR, a PI3K inhibitor. PI3K is involved in the insulin signaling cascade, leading to the increased survival of adipose cells by the activation of the IGF-1 pathway. EVs released by adipose tissue-derived stem cells from obese patients presented reduction proangiogenic potential mainly due to the abnormalities of EVs contents, such as a reduction in VEGF, MMP-2, and miR-126 [191]. This miRNA further modulates homeostasis and vascular integrity through the RAF1/ERK signaling pathway and also modulates the expression of SPRED1, which has inhibitory actions to RAF1. Thus, SPRED1 is increased in obesity and reduces angiogenesis and vascular integrity, which may contribute to endothelial dysfunction. Indeed, by blocking miR-126 in endothelial cells’ derived EV, the authors demonstrated that proangiogenic effects—endothelial cell migration and proliferation—were lost [192]. Interestingly, authors demonstrated that high-glucose reduces miR-126 expression.

MiR-21 is upregulated during the adipogenic differentiation of human adipose tissue-derived mesenchymal stem cells (MSCs), being associated with the TGF-β pathway [193], and was also shown to be a target for weight reduction in vivo [194]. MiR-148 was found to be overexpressed in adipose tissue of high fat-fed mice and obese human subjects, promoting adipogenesis in MSCs by targeting Wnt1 [187,195]. Increased expression levels of members of the let-7 family, miR-26a and the miR-320 family were found during adipocyte differentiation [196]. Interestingly, the overexpression of miR-320c in MSCs enhanced the adipogenic differentiation and accelerated formation of mature adipocytes by inhibiting the Runt-related transcription factor 2 (RUNX2) [186].

MiR-146b levels in fat tissue samples from overweight or obese groups were higher when compared to the lean group [197]. MiR-146b is highly expressed in mature adipocytes, and its expression varies with the stage of the adipocytes development. MiR-146b inhibits the in vitro proliferation of visceral pre-adipocytes and promotes their differentiation by the inhibition of the transcription factor KLF7, which is an inhibitor of adipogenesis [197]. KLF7 inhibits the expression of the adipogenic transcription factors C/EBPa and PPARc and adipocyte-marker genes, such as the AP2 gene. Furthermore, KLF7 regulates the adipocytokine gene expression in mature adipocytes, inhibiting insulin secretion in pancreatic β-cells and suppressing the hexokinase 2 gene expression in muscle cells, affect insulin sensitivity [198]. Interestingly, C/EBPa has been associated with pancreas b cell apoptosis induced by pro-inflammatory cytokines [199]. These data suggest that KLF7 has a role in the pathogenesis of T2D [200].

The analysis of samples of subcutaneous adipose tissue revealed the increased expression of miR-519, and the decreased expression of the predicted target peroxisome proliferator-activated-receptor α (PPARα), impairing FFA oxidation. In vitro studies demonstrated that miR-519d increased lipid accumulation during adipocyte differentiation. While miR-519d, miR-10b, miR-663, and miR-30d were found to be increased in the adipose tissue, miR-18a was underexpressed [201].

Inflammatory processes in obesity are closely linked to insulin resistance, T2D, and cardiovascular disease. TLR/NFκB (Toll like receptor/Nuclear Factor κB) signaling in monocytes is regulated by miR-181a, -181b, and -181d. In obese subjects, the decreased expression of these miRNAs was found in monocytes, which was reversed with body weight loss. Although these miRNAs were identified as regulators of the TLR/NFκB pathway, only miR-181a had a significant correlation with metabolic syndrome and coronary artery disease [202]. In the opposite direction, miR-223 seems to exert a suppressive effect on the inflammatory cascade in visceral adipose tissue macrophages, probably as a compensatory adaptation to the inflammatory status [203].

### 4.2. Other Circulating miRNAs in Obesity

The altered expression of different circulating miRNAs has been described in obese subjects. Comparing obese and normal weight individuals, three circulating miRNAs were found differentially expressed: miR-31-5p, miR-2355-5p, and miR-206 [204]. Serum levels of miR-138, miR-376a, and miR-503 distinguish obese subjects from obese-T2D and T2D subjects [159]. Another study reported increased serum levels of miR-152 and miR-17, and a decreased level of miR-138 in obese patients when compared to the controls [205]. Plasma samples were used for the evaluation of miRNA expression profile in subjects submitted to bariatric surgery. Increased levels of miR-222, miR-140-5p, and miR-142-3p and decreased levels of miR-532-5p, miR-125b, miR-130b, miR-221, miR-15a, miR-520c-3p, and miR-423-5p, were found in obese subjects, and showed a strong association with fat mass measures. Additionally, 14 circulating miRNAs were modulated upon surgery-induced weight loss [206]. The MiR-223 levels were lower in both overweight and obese subjects, when compared to normal-weight controls, and were increased after lifestyle change and weight loss [207]. In addition to miR-223, the circulating levels of miR-143 were also significantly lower in obese subjects [208].

### 4.3. Single Nucleotide Polymorphisms and miRNAs in Obesity

Obesity is considered a low-grade chronic inflammation state and studies investigating the role of cytokine gene polymorphisms have been investigated. IL-18, a pro-inflammatory cytokine member of the interleukin-1 family, is elevated in obesity and acts in conjunction with other cytokines, including IL-1, to activate the NFκB and transcription activator-1, modulating the expression of inflammatory genes [209,210,211,212]. The IL-18 actions depend on binding to its heterodimeric receptor IL-18R, composed by the IL-18R1 chain, with a binding function, and the IL18RAP chain, involved in signal transduction [213]. Martínez-Barquero et al. (2017) investigated the association between the IL18RAP gene polymorphisms, body mass index, and obesity [214]. They selected five SNPs in the IL18RAP that could change the miRNA-136 binding site. MiR-136 is associated with adipogenic differentiation and is involved in appetite control and energy homeostasis in hypothalamic neurons [215,216,217]. The rs7559479 G allele has a high association with obesity and the body mass index which augments the susceptibility to obesity. On the other side, the rs7559479 A allele was associated with an increased miR-136 binding to IL18RAP, enhancing IL-18 signaling.

As mentioned before, the genetic polymorphisms of the 3’-UTR in microRNA target genes may change the microRNA binding sites. Richardson et al. (2011) evaluated the association of SNPs and obesity, demonstrating the role of the creation of a new regulatory site to miRNA due to these SNP in disease development [218]. These studies demonstrated the association between obesity and SNPs at human PLIN4/S3-12, which is a member of the PAT (perilipin, adipose differentiation-related protein (ADRP), and tail-interacting protein of 47 kilodaltons-TIP47) Family lipid storage droplets (LSDs). It was described that a series of anthropometric characteristics found in obese people were modulated through a diet enriched in polyunsaturated fatty acids. The mechanism proposed is that *PAT* genes respond to a hyperlipidic diet at the transcriptional level, since polyunsaturated fatty acids are ligands of peroxisome proliferator-activated receptor (PPAR) whose response-elements (PPREs) are expressed in the promoter regions of PLIN1 and PLIN4 [219]. The results pointed to the relevance of the rs8887 A allele in creating a new binding site specifically to miR-522 that promoted the reduction in PLIN4 protein levels. These finding demonstrated the effects of the association of genetic factors with environmental factors in the susceptibility to obesity development. 

Another key regulatory element in the lipid metabolism is a serine hydrolase known as Butyrylcholinesterase (BChE; EC 3.1.1.8), which is related to metabolic syndrome risk in obese patients [220]). In these individuals, the BChE expression is higher than in lean subjects, probably due to the elevated free fatty acid (FFA) circulating levels [221]. *BChE* gene polymorphisms have been reported before in, for example, the study conducted by Lima and colleagues (2013), in which the role of the 1914G allele of *BChE* gene in metabolism was investigated [222]. This allele represents a site of different microRNAs, including hsa-miR-498 and hsa-miR-662, regulating the BChE expression and activity, leading to alterations in different metabolic pathways and contributing to the development of obesity. These findings demonstrate that SNPs in a variety of genes involved in the metabolism may affect homeostasis, being exacerbated by environmental factors.

## 5. Overlapping miRNAs in SAH, T2D, and Obesity

**Figure 4:**
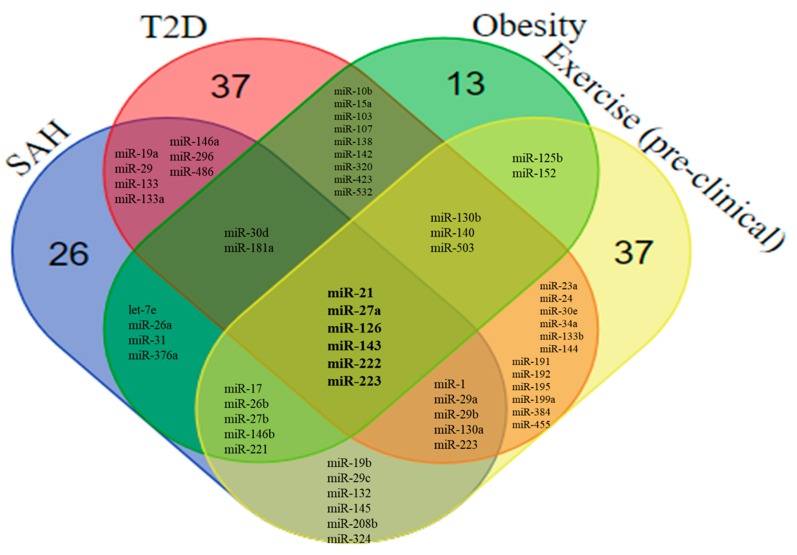
A Venn diagram of hypertension, diabetes, obesity, and their overlapping with exercises (pre-clinical). The Venn diagram was used to identify overlapping and non-overlapping miRNAs in the analysis of studies on the microRNA profile of expression in cardiometabolic diseases and pre-clinical studies on exercise training.

**Figure 5:**
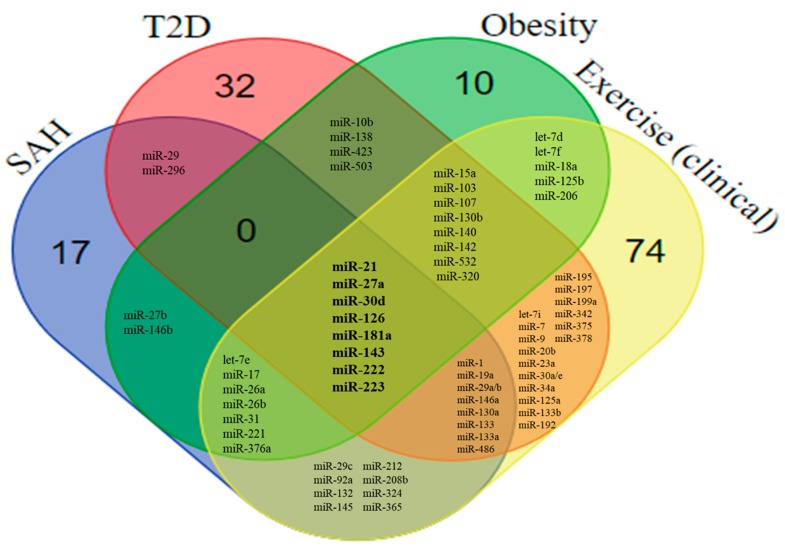
A Venn diagram of hypertension, diabetes, obesity, and their overlapping with exercises (clinical). The Venn diagram was used to identify overlapping and non-overlapping miRNAs in the analysis of studies on the microRNA profile of expression in cardiometabolic diseases and clinical studies on exercise training.

After the review of miRNAs associated with SAH, T2D, and obesity, it was possible to find 144 miRNAs with an altered expression in experimental and clinical studies. Although considerable variability was found in terms of design, sample, and methodology, it was possible to identify 16 miRNAs with an altered expression that overlapped between T2D and SAH, 19 between T2D and obesity, and 15 between obesity and SAH. We also found 8 miRNAs—miR-21, miR-27a, miR-30d, miR-126, miR-143, miR-181a, miR-222, and miR-223—that overlapped between the three diseases (Figure 4 and Figure 5).

MiR-21 was upregulated in SAH, T2D, and obesity, both in experimental and in clinical studies, suggesting a potential role in the common aspects of the disease pathogenesis. MiR-21 is highly expressed in VSMCs, endothelial cells, cardiomyocytes, and cardiac fibroblasts, acting on different targets, including PDCD4 (programmed cell death protein 4), PTEN (phosphatase and tensin homolog protein), SPRY1 (protein sprouty homolog 1), and SPRY2 (protein sprouty homolog 2) [19], being associated with apoptosis, inflammation, and participating in VEGF and TGF-β signaling pathways. MiR-21 has also a role in the promotion of cardiac hypertrophy [223].

The underexpression of miR-126 is consistently reported in the context of SAH, T2D, and obesity in both experimental and clinical studies. Under physiological conditions, miR-126 is highly expressed in endothelial cells, being involved in angiogenesis and with anti-inflammatory actions. MiR-126 regulates the expression of the chemokine (C-C motif) ligand 2 (CCL2) [35], which has been associated with the recruitment of inflammatory cells and insulin resistance [224]. Likewise, VCAM-1, which is involved in leukocyte-endothelial cell adhesion and may play a role in the development of atherosclerosis, is down-regulated by miR-126 under physiological conditions. By reducing the VCAM-1 expression, miR-126 led to a decreased vascular inflammation due to a reduced interaction between leukocytes and endothelial cells [54]. Moreover, reduced miR-126 expression impairs angiogenesis and vascular integrity through the inhibition of PI3KR2 [51].

Data from clinical studies revealed the increased expression of circulating miR-222 in SAH, T2D, and obesity. MiR-222 participates in several physiological functions in the cardiovascular system and an increasing body of data has pointed to a protective role played by miR-222, promoting angiogenesis and regulating physiological functions of cardiomyocytes [225].

MiR-223 was found to be increased in the adipose tissue in obesity, working as a compensatory anti-inflammatory molecule, but with a reduced expression in the circulation of obese and T2D subjects [203]. Additionally, the experimental data suggest that miR-223 could be increased in SAH, but there are no clinical validation studies available so far.

Circulating levels of miR-143, which targets angiotensin converting enzyme (ACE), were reduced in hypertensive, obese, and T2D subjects in one study. There is evidence, however, that the tissue overexpression of miR-143 contributes to insulin resistance in T2D [226]. The tissue overexpression of miR-143 was also found in different experimental models of diabetes in mice, with a negative regulation of insulin-mediated AKT activation [227].

Different studies have reported reduced circulating levels of miR-181a both in T2D and obesity, while increased levels have been reported for hypertensive patients [89]. The opposite pattern was found for miR-30d, with increased circulating levels in T2D and obesity, and reduced levels in SAH [99,141,201]. MiR-27a was increased and associated with hyperglycemia and metabolic syndrome in T2D patients, also playing a role in adipogenesis and in the regulation of blood pressure [228].

Currently, it is not completely understood whether the reported miRNAs are involved in the early or advanced aspects of the disease pathogenesis. Further studies are needed in order to define the roles of individual miRNAs and their targets in different disease settings, tissues, and diseases. MiRNAs with differential expression could potentially be applied as biomarkers for diagnosis, prognosis, risk stratification, or even be therapeutically targeted. Nevertheless, this would require larger validation studies, considering that the studies performed vary significantly in terms of design.

## 6. Exercise and Cardiovascular Protection 

Exercise training is a classical recommendation for patients with cardiometabolic diseases and The American Heart Association recommends at least 30 min of aerobic exercise five days a week in order to promote cardiovascular risk reduction [229]. Aerobic exercise training is able to ameliorate dyslipidemia, insulin resistance, endothelial function, and inflammatory status [230]. It is well known that exercise training exerts beneficial effects in healthy [231] and in individuals with SAH [232], T2D [233], obesity [234,235], cancer [236], coronary heart disease [237], and chronic heart failure [238].

Aerobic exercise training induces a physiological eccentric cardiac hypertrophy, while resistance training promotes a physiological concentric hypertrophy [239]. The experimental data suggest that exercise training may counteract the pathological cardiac remodeling, decreasing fibrosis, and improving heart function [240,241]. Anti-remodeling actions of exercise training were previously demonstrated in subjects with heart failure [242] and also in experimental models of myocardial infarction and diabetes [241,243]. Little is known about the mechanisms and molecular pathways involved in the anti-remodeling actions of exercise training. One of the mechanisms suggested to explain the exercise-mediated regulation of cellular homeostasis is through modulation in the miRNA expression profile.

### 6.1. Altered miRNA Expression Induced by Exercise Training

Cellular homeostasis is affected by exercise, with influences in the circulating miRNA signature. These alterations in the circulating miRNA profile are dynamic and change significantly during the acute response and chronic adaptation to exercise [244]. Different cell types may be involved in the release of miRNAs into the circulation immediately after acute exercise, which probably reflects exercise-induced tissue stress or damage [245]. Moreover, tissue analyses have clearly demonstrated the effects of different types of exercise in the miRNA profile in the vasculature, heart, and skeletal muscles.

In addition to the study of miRNA dysregulation in SAH, T2D, and obesity, the influence of exercise training in the expression of miRNAs in pre-clinical and clinical studies was also reviewed (Tables S1 and S2). Considerable variation in the results was found, and this can be attributed to the variation in methods, protocols and other aspects of individual variation, such as age, sex, ethnicity, and genetic background.

### 6.2. Overlaps Between the miRNA Profile in SAH, T2D, Obesity, and Exercise

The beneficial effects of exercise training in miRNA-mediated gene regulation in the population of patients with SAH, T2D, and obesity are currently unexplored since the majority of clinical studies focus on healthy subjects. Identification of miRNAs regulated by exercise in diseased subjects would be important to understand the dynamic alterations and protective roles exerted by exercise training in the context of cardiovascular diseases. In our review, 76 miRNAs with altered expression induced by exercise were described in the experimental models, while 141 were found in clinical studies (Table S1). By comparing the expression profile of these miRNAs with the miRNAs reviewed in the context of SAH, T2D, and obesity, we found significant overlaps (Figure 4 and Figure 5). Among the miRNAs evaluated in the experimental models, we found miR-21, miR-27a, miR-126, miR-143, miR-222, and miR-223 in common with all three diseases (Figure 4). In addition to these miRNAs, miR-30d and miR-181a were also reported to be altered in clinical studies (Figure 5).

Pre-clinical studies reported that miR-21 is increased in the heart after a chronic adaptation to exercise [246,247]. Non-pathological left ventricular hypertrophy is a normal adaptation of exercise training and increased miR-21 expression may be involved in this process [248]. Meanwhile, in clinical studies, there is considerable variability regarding the circulating levels of miR-21 in healthy subjects submitted to different protocols of exercise training. Radom-Azik and colleagues reported increased miR-21 levels in the PBMCs during the acute response to cycle ergometer exercise [249], while Baggish and colleagues reported increased circulating miR-21 in serum samples during both the acute and chronic response after rowing training [250,251]. In chronic heart failure subjects, however, acute exhaustive exercise-induced an increase in the serum levels of miR-21, suggesting its potential role in the adaptation to exercise [252]. An opposite regulation of miR-21 was reported in the acute phase and chronic response to exercise training. Baggish and colleagues found increased levels of miR-21 in the acute phase [250], while Nielsen and colleagues reported the underexpression of miR-21 in the plasma after a chronic adaptation to cycling ergometer training [244]. This variability may also be explained by the diversity of exercise training protocols, the type of samples used in the analyses, and also by the small sample sizes, with no more than 32 subjects included in each study.

MiR-27a and miR-27b were reduced during adipogenesis [181], while exercise increased their expression [239,253]. The increased expression of miR-27a and miR-27b could be involved in the control of body mass [254]. The gene coding PPARγ, which plays roles in the adipocyte differentiation, is a target of miR-27a [254]. Therefore, its inhibition promoted by the increased expression of miR-27a following exercise training could be involved in the control of body mass. Pre-clinical studies reported that miR-27a is increased in skeletal and cardiac muscle after acute and chronic training [255,256]. In a clinical study, the evaluation of circulating levels of miRNAs demonstrated decreased plasma levels of miR-27a in obese subjects in the acute and chronic response of exercise training [230]. Increase miR-27a expression was found in rats after swimming training [256].

Pre-clinical studies have reported increased miR-126 expression in the myocardium after a chronic exercise training adaptation [257,258]. Clinical studies have focused on the acute response to different protocols of exercise training in healthy subjects, where decreased levels of miR-126 were reported in PBMCs [249,259]. Two studies, however, reported increased miR-126 levels in the plasma of healthy men after marathon runs [251,260]. The induction of increased levels of miR-126 by exercise could counteract the underexpression found in obesity, SAH, and T2D.

MiR-143 was found to be reduced in the heart, experimentally, after a chronic adaptation to exercise training [261]. In clinical studies, however, aerobic training induced an acute increase in miR-143 circulating levels [244,262]. The miR-143/miR-145 gene cluster, which negatively regulates the *ACE* gene expression, is a regulator of the contractile phenotype of VSMCs [10]. In contrast, in exercise, these miRNAs presented an increased expression [244,246]. The difference status in their expression indicates that this could be one important mechanism of blood pressure control induced by exercise. Experimental studies suggest that ACE2 is important for cardiovascular function [263] and, in spontaneously hypertensive rats, chronic aerobic exercise training was associated with increased levels of miR-27a (targeting ACE) and decreased levels of miR-143 (targeting ACE2), contributing to blood pressure regulation [264]. Aerobic exercise training could induce a nonpathological left ventricular hypertrophy, altering the expression of miRNAs that regulate RAAS genes [256].

Interestingly, miR-21 and miR-143 showed a different response when evaluated after acute and chronic training. Increased levels of miR-143 were observed 1 h after acute exercise [244] and decreased in chronic training [256]. Increased levels of circulating miR-21 were observed in response to acute aerobic exercise and decreased levels in chronic training [244]. It is possible that the acute increase in miR-21 may be involved in inflammation [265], while regular exercise exerts long-term anti-inflammatory effects, correlating with decreased miR-21 levels in the chronic adaptation phase [244].

It was demonstrated that miR-126 increased after 10 weeks of training based on swimming [258], which could contribute to the report of balanced angiogenic and apoptotic factors and the normalization of VEGF, eNOS, and PI3KR2 levels observed after exercise training in hypertensive rats [266]. The MiR-126 expression has been demonstrated to be associated with angiogenesis, anti-inflammatory response, and atherosclerosis [267]. MiR-126 negatively regulates the expression of the *CCL2* gene [35], which is involved in the development of the obesity-induced inflammation and insulin resistance in the skeletal muscle [224]. MiR-126 also reduces vascular inflammation by decreasing interactions between leukocytes and endothelial cells and diminishing the production of inflammatory mediators [54,268].

The cardiac expression of miR-222 was increased in the acute response after exercise in an experimental study [269] and was also increased in the serum of both the acute and chronic phase after exercise training in healthy subjects [250]. Interestingly, miR-222 was also found to be acutely increased in plasma samples of subjects with chronic heart failure submitted to aerobic training by a bicycle ergometer test [269]. MiR-223 is also increased in the circulation of healthy subjects during the acute response after exercise training, while upregulation was described in the heart tissue after chronic aerobic training in a preclinical study [248,262,270].

### 6.3. SAH vs. Exercises: miRNAs Differentially Expressed 

**Figure 6:**
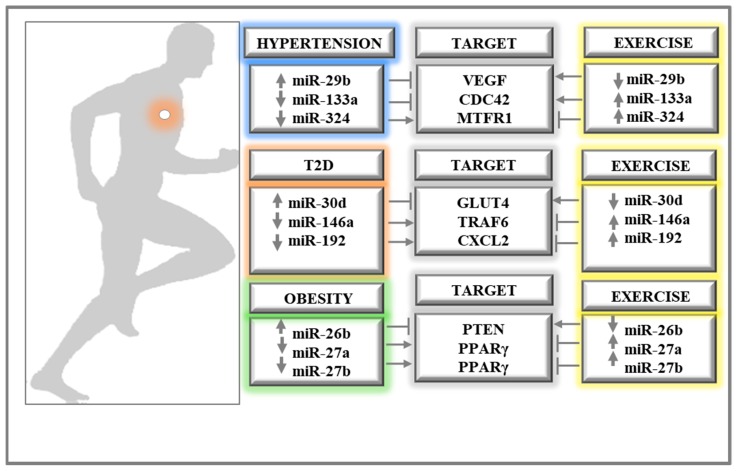
The opposite regulation of miRNA expression after exercises and their targets. The scheme illustrates examples of miRNAs with differential expression reported for diseases and exercise training. The respective gene target is also indicated. ↑ = upregulated, ↓ = downregulated.

Aerobic training has been implicated as an important nonpharmacological treatment for hypertension [266]. The protective effects of physical training in vascular pathological alterations have been reported to occur through the regulation of miRNAs. Different miRNAs with descriptions of opposite expression profiles in SAH and exercises were described in the literature and are described below (Figure 6).

Increased levels of miR-146a and miR-126 along with reduced miR-155 levels were found on the aorta of mice with atherosclerosis after 12-week aerobic exercise [271]. Increase levels of miR-16, miR-21, and miR-126 were found in the muscles of hypertensive rats after 10 weeks of swimming, correlating with a balance between angiogenic and apoptotic factors [272]. Interestingly, miRNAs regulating the gene of ACE were differentially expressed in both hypertension and exercise, like the exercise-induced increase of miR-27a (targeting ACE) and miR-155 (targeting AT1R) and the decrease of miR-143 (targeting ACE2) [264]. Plasma levels of miR-92 increased in hypertensive patients, correlating with the blood pressure levels [96], while it was described to be reduced during chronic adaptation to exercise training [244].

MiR-29b was highly expressed in SAH [97] and indirectly regulates the expression of the *VEGFA* and *COL1A2* genes. Interestingly, exercise reduces miR-29b expression [231], suggesting that this process may be involved in the amelioration of the endothelial function promoted by exercise.

MiR-19b, which was found downregulated in SAH, was reported to be increased by exercise training in pre-clinical and clinical studies [273,274]. Other miRNAs presented opposite expressions between SAH and exercise. This is the case of miR-324, which was found reduced in SAH [99] and increased by aerobic exercise training, both experimentally [253] and in human subjects [275]. This finding could be associated with cardioprotective actions since miR-324 regulates mitochondrial fission and apoptosis by targeting Mtfr1, leading to a suppression of cardiomyocyte death and myocardial infarction [276].

The downregulation of miR-133a is involved in the process of cardiac hypertrophy since miR-133a indirectly targets anti-hypertrophic genes [277]. The MiR-133a expression levels were reduced in SAH, while circulating levels were increased in healthy subjects after exercise training [244,251,278,279]. Interestingly, the miR-133a overexpression prevents cardiac fibrosis in a mouse model of diabetic cardiomyopathy [280]. In contrast, miRNA-22 is overexpressed in SAH, being involved in the sympathetic nervous system hyperactivation, and reduced by exercise [95,273].

### 6.4. T2D versus Exercises: miRNAs Differentially Expressed

Different miRNAs with descriptions of opposite expression profiles in T2D and exercises were described (Figure 6). MiR-192 was found to be reduced in T2D patients [146] and increased in heart samples of rats after chronic adaptation to exercise [253]. Moreover, increased levels of miR-192 were found after acute exercise training in humans [259]. This miRNA regulates the *CXCL2* gene, which is part of a superfamily of chemokines associated with inflammation and, therefore, could contribute to the inflammatory response associated with T2D [281]. Since miR-192 can inhibit CXCL2 activity, this process could be involved in the anti-inflammatory properties of exercise, which can be beneficial in T2D patients.

Corroborating with this hypothesis, other miRNAs involved in the regulation of inflammation are differentially expressed between T2D and exercise training, including miR-146a. Clinical studies have demonstrated that circulating levels of miR-146a can be found either upregulated or downregulated in T2D patients [282,283], while two independent reports described that aerobic exercise training can increase miR-146a levels [250,251]. Resistance exercise training, however, was found to acutely decrease miR-146a expression in healthy subjects [284]. One of the targets of miR-146a is *TRAF6*, a member of the TNF receptor-associated factor protein family and mediates the signal transduction of TNF receptors. Additionally, the protein encoded by *TRAF6* gene is a signal transducer and participates in the pathway that activates IkappaB kinase (IKK) in response to proinflammatory cytokines [285]. Notably, increased levels of miR-146a induced by exercise may contribute to the modulation of the chronic inflammatory response in T2D. MiR-24 is another miRNA with anti-inflammatory actions that was found downregulated in the circulation of T2D patients [144,286]. In an experimental study, exercise training was associated with increased cardiac expression of miR-24 [253].

MiR-144 is overexpressed in T2D and regulates the IRS, the initiator of insulin signaling [127,128]. This is one of the molecular mechanisms leading to insulin resistance. Studies diverge about the exercise-induced response, with reports of both increased and decreased expression of miR-144 [246,253]. Further studies with clinical validations are needed to analyze the effects of physical exercise on the expression of miR-144. MiR-9 negatively affects the process of insulin secretion by targeting Onecut2, and has been found to be overexpressed in T2D, being downregulated in the skeletal muscle, in the acute response to ergometer cycle [287]. MiR-384 is involved in insulin resistance, impairing the GLUT4 translocation to the cell surface by targeting the PI3K/AKT axis, and was found to be increased in T2D and decreased in the heart after chronic exercise training in rats [253].

### 6.5. Obesity vs. Exercises: miRNAs Differentially Expressed 

Other miRNAs were found with opposite expression between obesity and exercise training (Figure 6), including miR-26a, miR-27b, and miR-206. The MiRNA-26 family is also important to adipogenesis and their inhibition reduces lipid accumulation, while their overexpression increases it [183,184,186,187]. Exercise can reduce the expression of miR-26a after aerobic [231] and resistance exercises [288]. MiR-27b targets PPARγ, being reduced in obesity and increased after exercise [253,256]. MiR-206, which was shown to block adipogenesis [289], is reduced in the circulation of obese subjects, and increased after marathon runs in healthy subjects [290].

## 7. Conclusions

MiRNAs are involved in the pathogenesis of cardiometabolic diseases and common regulatory pathways can be identified in SAH, T2D, and obesity. Exercise training modulates the expression of several miRNAs—both in the short-term and in long-term—which act by regulating targets that affect different key cellular and molecular processes, frequently in the opposite direction to that described to be involved in the pathogenesis of cardiometabolic diseases. Further validation studies are needed to evaluate the cellular and molecular effects of exercise training in these diseases and to confirm the ability of exercise training to ameliorate the altered miRNA expression profile found in patients with cardiometabolic diseases, especially regarding long-term effects. Additionally, the identification of miRNAs beneficially modulated by exercise training can contribute to the development of new drugs and therapies for the treatment of SAH, T2D, and obesity.

## Supplementary Materials

Supplementary materials can be found at http://www.mdpi.com/1422-0067/19/11/3608/s1. References [231,234,241,244,246,247,248,249,250,251,253,254,255,256,257,258,259,260,261,262,269,273,274,275,278,279,284,287,288,290] are cited in the supplementary materials.
