# Supplementary material for: Exercise Training-Induced Changes in MicroRNAs: Beneficial Regulatory Effects in Hypertension, Type 2 Diabetes, and Obesity
Source: Int J Mol Sci. 2018 Nov 15;19(11):3608. doi: 10.3390/ijms19113608 (PMC6275070; doi:10.3390/ijms19113608)
Supplement: Supplementary file 1 [file ijms-19-03608-s001.zip › Supplementary Materials.docx]

**Table S1.** MicroRNAs expression after exercise in pre-clinical studies.

|  | MicroRNAs in Exercise | | | | |  |
| --- | --- | --- | --- | --- | --- | --- |
| MicroRNAs | Targets | Source | Types of Exercises | Reference |  |  |
| In vivo experimental models | | | | | | |
| 🡩 miR-27a, miR-155    🡫 miR-143 | ACE, AT1R  ACE2 | Heart samples | Chronic Adaptation  (12 weeks)  Wistar-Kyoto rats  Exercise training on treadmill | [234] |  |  |
| 🡩 miR-17-3p | TIMP-3  PTEN | Heart samples | Chronic Adaptation  (21 days total)  C57Bl/6 mice  Ramp swimming training model  Voluntary wheel training | [291] |  |  |
| 🡩 miR-222 | HIPK1 | Heart samples | Chronic Adaptation  (3 weeks total)  Ramp swimming model  Voluntary wheel training | [269] |  |  |
| 🡩 miR-19b, miR-30e, miR-133b, miR-208a  🡫 miR-99b, miR-100, miR-191a, miR-22, miR-181a | IGF1  PI3/AKT/mTOR  MAPK  p53 | Heart samples  Plasma | Chronic Adaptation  (8 weeks total)  Wistar albino rats  Swimming training  (90 min, twice daily)  5% caudal body weight workload | [273] |  |  |
| 🡩 miR-146a, miR-126  🡫 miR-155 | TRAF6 | Heart samples | Chronic Adaptation  (12 weeks)  Voluntary wheel training Exercise training on treadmill | [241] |  |  |
| 🡩 miR-29a, miR-101a | TGF-β  fos  COL1A1 | Heart samples | Chronic Adaptation  (8 weeks total)  Sprague dawley rats  Intermittent run exercise  (60 min, 5d/ week) | [292] |  |  |
| 🡩 miR-27a, miR-27b  🡫 miR-143 | ACE  ACE2 | Heart samples | Chronic Adaptation  (10 weeks total)  Wistar rats  Swimming training  Moderate and high volume  (60 min, 5d/ week)  5% caudal body weight workload | [256] |  |  |
| 🡩 miR-126 | PI3KR2 | Heart samples  Plasma | Chronic Adaptation  (10 weeks total)  Zucker rats  Swimming training  (60 min, 5d/ week) | [258] |  |  |
| 🡫 miR-214 | SERCA2A | Heart samples | Chronic Adaptation  (8 weeks total)  Wistar rats  Resistance training  (5d/ week; 80% of 1 RM) | [293] |  |  |
| 🡩 miR-1  🡫 miR-214 | NCX  SERCA2A | Heart samples | Chronic Adaptation  (10 weeks total)  Wistar rats  Swimming training  (60 min, 5d/ week; mild-intensity long-period)  3% caudal body weight workload | [294] |  |  |
| 🡩 miR-29c  🡫 miR-1, miR-133a, miR133b | COL1A1  COL3A1 | Heart samples | Chronic Adaptation  (10 weeks total)  Wistar rats  Swimming training  (60 min, 5d/ week)  5% caudal body weight workload | [295] |  |  |
| 🡩 miR-126 | SPRED1  PI3KR2 | Heart samples | Chronic Adaptation  (10 weeks total)  Wistar rats  Swimming training  (60 min, 5d/ week)  5% caudal body weight workload | [257] |  |  |
| 🡩 miR-21, miR-144, miR-145  🡫 miR-124 | PTEN  PIK3A  TSC2 | Heart samples | Chronic Adaptation  (8 weeks total)  Wistar rats  Swimming training  (60 min, 5d/ week)  5% caudal body weight workload | [246] |  |  |
| 🡩 miR-336-5p, miR-130b-5p, let7d-3p, miR-466c-5p, miR-324-3p, miR-146b-5p, miR-132-3p, miR-21-5p, miR-187-3p, miR-29b-5p, miR-324-5p, miR-214-5p, miR-140-5p, miR-152-5p, miR-99b-5p, miR-130a-5p, miR-455-5p, miR-27b-3p, miR-23b-3p, miR-652-5p, miR-199a-3p, miR-223-5p, miR-421-3p, miR-27a-5p, miR-24-5p, miR-34a-3p, miR-140-3p, miR-125b-5p, miR-145a-5p, miR-192-5p, miR-139-5p, miR-199a-5p, miR-674-3p, miR-191-5p, miR-28-3p, miR-195-5p, miR-598, miR-429, miR-224, miR-425, miR-221  🡫 miR-701-5p, miR-220, miR-144-3p, miR-694, miR-485-3p, miR-136-5p, miR-384-3p, miR-376c-3p, miR-208b-3p, miR-411-3p, miR-141-5p, miR-1894-3p, miR-9a, miR-687, miR-451-5p | TNF-α  COL1A1  MMP9  PTEN  AKT1  AMPK  p38  BCL2 | Heart samples | Chronic Adaptation  (10 weeks total)  Wistar rats  Aerobic run training  (Progressive intensity; 5d/ week) | [253] |  |  |
| 🡩 miR-503, miR-465b-5p, miR-542-3p  🡫 miR-652 |  | Heart samples | Chronic Adaptation  (6 weeks total)  C57Bl6 mice  Swimming training  (3x30 min, each week) | [296] |  |  |
| 🡫 miR-26b, miR-143 | IGF1R  GATA-4  NFAT1C  GSK3B | Heart samples | Chronic Adaptation  (35 days total)  Balb/c mice  Aerobic metal wheels training | [261] |  |  |
| 🡩 miR-21, miR-30b  🡫 miR-1 | BCL-2  p53  PDCD4  DRP-1 | Heart samples | Chronic Adaptation  (8 weeks total)  Swimming training  (2 times/day, 5d/ week) | [247] |  |  |
| 🡩 miR-23a, miR-27a | PTEN  Casp7  FoxO1 | Skeletal muscle samples | Acute Response  Resistance exercise  (Muscle overload) | [255] |  |  |
| 🡩 miR-29c  🡫 miR-1 | COL1A1  COL3A1 | Heart samples | Chronic Adaptation  (10 weeks total)  Zucker rats  Swimming training  (60min, 5d/ week, with 4% of body weight workload) | [297] |  |  |

**Table S2.** MicroRNAs expression after exercise in clinical studies.

|  | MicroRNAs in Exercise | | | |
| --- | --- | --- | --- | --- |
| MicroRNAs | Targets | Source | Types of Exercises | Reference |
| Clinical studies | | | | |
| 🡩 miR-126, miR-133 | CPK | Plasma | Acute Response  Single symptom-limited spiroergometry test  Cycling 4 h at 70% of anaerobic threshold  Marathon run  Eccentric resistance exercise | [260] |
| 🡫 miR-486 | PTEN | Serum | Acute Response  Cycle ergometry 60 min.  at 70% VO2max  Chronic Adaptation  (4 weeks total)  Systematic—cycling at 70% VO2max  (3 x30 min/ week) | [298] |
| 🡩 miR-1, miR-126, miR-133a, miR-134, miR-146a, miR-208a, miR-499-5p | CPK  NT-proBNP  hsCRP | Plasma | Acute Response  Marathon run  Immediately after run  (decreased after 24 h) | [251] |
| 🡩 miR-1, miR-133a, miR-206, miR-208b, miR-499 |  | Plasma | Acute Response  Marathon run  Immediately after run | [290] |
| 🡩 miR-1, -133a, -206 |  | Plasma | Acute Response  Marathon run  Immediately after run | [278] |
| 🡩 let-7d-3p, let-7f-3p  miR-29a-3p, miR-34a-5p, miR-125b-5p  miR-132-3p, miR-143-3p,  miR-148a-3p, miR-223-3p, miR-223-5p  miR-424-3p, miR-424-5p |  | Serum | Acute Response  Marathon run  Immediately after run  (decreased after 24 h) | [262] |
| 🡩miR-1, -30a, -133a  🡫miR-26a, -29b |  | Plasma | Acute Response  Marathon run  Immediately after run  (decreased after 24 h)  Immediately after run | [231] |
| 🡩 miR-1, -133a, -206 |  | Plasma | Acute Response  Marathon run  Immediately after run  (decreased after 24 h) | [279] |
| 🡩miR-1, miR-133a, miR-133b, miR-139-5p, miR-143, miR-145, miR-223, miR-330-3p, miR-338-3p, miR-424  🡫 miR-30b, miR-106a, miR-146, miR-151-3p, miR-151-5p, miR-221, miR-652, let-7i  🡩 miR-103, miR-107,  🡫 miR-21, miR-25, miR-29b, miR-92a, miR-133a,  miR-148a, miR-148b, miR-185,  miR-342-3p, miR-766, let-7d |  | Plasma | Acute Response  cycle ergometry test  at 65% Pmax  1-3 hs after exercise  Immediately after exercise  Chronic Adaptation  (12 weeks total)  Systematic endurance cycle  ergometry training,  3-5 days after training | [244] |
| 🡩miR-1, miR-133a, miR-133b, miR-206  miR-485-5p, miR-509-5p, miR-517a  miR-518f, miR-520f, miR-522, miR-553, miR-888 |  | Plasma | Acute Response  High intensity interval exercise  Immediately after  Vigorous intensity continuous exercise  Immediately after | [299] |
| 🡩 miR-181b, miR-214  🡩 miR-1, miR-133a, miR-133b, miR-208b |  | Plasma | Acute Response  Uphill treadmill test (concentric)  Immediately after  Downhill treadmill test (eccentric)  2-6 hs after exercise | [300] |
| 🡩miR-149  🡫 miR-146a, miR-221 |  | Serum | Acute Response  Resistance exercise  (bench press and leg press)  3 days after exercise | [284] |
| 🡩 miR-1, miR-133a, miR-133b, miR-206, miR-208b, miR-499 |  | Plasma | Chronic Adaptation  (5 months total)  Systematic resistance training  36-72 hs after training | [301] |
| 🡩 miR-1, miR-133a, miR-133b, miR-181a  🡫 miR-9, miR-23a, miR-23b, miR-31  🡩 miR-1, miR-29b | HDAC4  NRF1 | Skeletal muscle samples | Acute Response  (Cycle ergometer, 60 min, 70% VO2peak)  Chronic Adaptation  (10 days total)  Cycling | [287] |
| 🡩 miR-136, miR-200c, miR-376a, miR-377, miR-499b, miR-558  🡫 miR-28, miR-30d, miR-204, miR-330, miR-345, miR-375, miR-449c, miR-483, miR-509, miR-520a, miR-548an, miR-628, miR-653, miR-670, miR-889, miR-1245a, miR-1270, miR-1280, miR-1322, miR-3180 |  | Skeletal muscle samples | Chronic Adaptation  (12 weeks total)  Resistance training  (8x5 leg press repetitions, 80% of 1 RM) | [302] |
| 🡩miR-451  🡫 miR-26a, miR-29a, miR-378 |  | Skeletal muscle samples | Acute Response  Resistance exercise  (High vs. Low responders) | [288] |
| 🡩miR-125a, miR-145, miR-181b, miR-193a, miR-197, miR-212, miR-223, miR-340, miR-365, miR-485, miR-505, miR-520d, miR-629, miR-638, miR-939, miR-940, miR-1225, miR-1238  🡫 miR-let-7i, miR-16, miR-17, miR-18a, miR-18b, miR-20a, miR-20b, miR-22, miR-93, miR- 96, miR-106a, miR-107, miR-126, miR-130a, miR-130b, miR-151, miR-185, miR-194, miR-363, miR-660 |  | Serum | Acute Response  Cycle ergometer exercise  (10x2min bouts, 1min rest interval between each bout, 76% VO2peak) | [303] |
| 🡩miR-7, miR-15a, miR-21, miR-26b, miR-132, miR-140, miR-181a, miR-181b, miR-181c, miR-338, miR-363, miR-939, miR-940, miR-1225  🡫 miR-let-7e, miR-23b, miR-31, miR-99a, miR-125a, miR-125b, miR-126, miR-130a, miR-145, miR-151, miR-199a, miR-199b, miR-221, miR-320, miR-451, miR-486, miR-584, miR-652 |  | PBMC | Acute Response  Cycle ergometer exercise  (10x 2min bouts, 1min rest interval between each bout, 76% VO2peak) | [249] |
| 🡩miR-let-7f, miR-21, miR-29c, miR-223  🡫 miR-let-7f, miR-21, miR-29c, miR-223 |  | PBMC | Chronic Adaptation  (18 weeks)  Running exercise  (3x/week, 60 min) | [248] |
| 🡩miR-7, miR-29a, miR-29b, miR-29c, miR-30e, miR-142, miR-192, miR-338, miR-363, miR-590  🡫 miR-let-7e, miR-126, miR-130a, miR-151, miR-199a, miR-221, miR-223, miR-326, miR-328, miR-652 |  | PBMC | Acute Response  Cycle ergometer exercise  (10x 2min bouts, 1min rest interval between each bout, 77% VO2peak) | [259] |
| 🡩miR-15a, miR-29b, miR-29c, miR-30e, miR-140, miR-324, miR-338, miR-362, miR-532, miR-660  🡫 miR-23b, miR-130a, miR-151, miR-199a, miR-221 |  | Serum | Acute Response  Cycle ergometer exercise  (10x 2min bouts, 1min rest interval between each bout, 82% VO2max) | [275] |
| 🡩miR-1, miR-486, miR-494 |  | Serum | Acute Response  Aerobic exercise  (Endurance athletes, runners, cyclists and triathletes) | [304] |
| 🡩 miR-21, miR-146a, miR-221, miR-222  🡩 miR-20a, miR-21, miR-146a, miR-221, miR-222 |  | Serum | Acute Response  Cardiopulmonary exercise test  Chronic Adaptation  (90 days)  Rowing training, 5Km, 1-3 h per session, 20-24 strokes/min) | [250] |
| 🡩 miR-376a  🡫 miR-16, miR-27a, miR-28 |  | Plasma | Chronic Adaptation  (5 months total)  Aerobic run exercise training  (4 days/week) | [305] |
| 🡩 miR-19a, miR-19b, miR-20a, miR-26b, miR-143, miR-195 | p-AKT  p-S6K1 | Serum | Acute Response  Resistance exercise  (3x bilateral knee extension and leg press, 10 rep, 80% of 1 RM) | [274] |
| 🡩 miR-222 | HIPK1 | Plasma | Acute Response  Heart failure patients  Bicycle Ergometry Test | [269] |

References

291. Shi, J.; Bei, Y.; Kong, X.; Liu, X.; Lei, Z.; Xu, T.; Wang, H.; Xuan, Q.; Chen, P.; Xu, J.; et al. miR-17-3p contributes to exercise-induced cardiac growth and protects against myocardial ischemia-reperfusion injury. *Theranostics* **2017**, *7*, 664–676, doi:10.7150/thno.15162.

292. Xiao, L.; He, H.; Ma, L.; da, M.; Cheng, S.; Duan, Y.; Wang, Q.; Wu, H.; Song, X.; Duan, W.; et al. Effects of miR-29a and miR-101a expression on myocardial interstitial collagen generation after aerobic exercise in myocardial-infarcted rats. *Arch. Med. Res.* **2017**, *48*, 27–34, doi:10.1016/j.arcmed.2017.01.006.

293. Melo, S.F.S.; Barauna, V.G.; Júnior, M.A.C.; Bozi, L.H.M.; Drummond, L.R.; Natali, A.J.; de Oliveira, E.M. Resistance training regulates cardiac function through modulation of miRNA-214. *Int. J. Mol. Sci.* **2015**, *16*, 6855–67, doi:10.3390/ijms16046855.

294. Melo, S.F.S.; Barauna, V.G.; Neves, V.J.; Fernandes, T.; Lara, L. da S.; Mazzotti, D.R.; Oliveira, E.M. Exercise training restores the cardiac microRNA-1 and -214 levels regulating Ca^2+^ handling after myocardial infarction. *BMC Cardiovasc. Disord.* **2015**, *15*, 166, doi:10.1186/s12872-015-0156-4.

295. Soci, U. P.R.; Fernandes, T.; Hashimoto, N.Y.; Mota, G.F.; Amadeu, M.A.; Rosa, K.T.; Irigoyen, M.C.; Phillips, M.I.; Oliveira, E.M. MicroRNAs 29 are involved in the improvement of ventricular compliance promoted by aerobic exercise training in rats. *Physiol. Genomics* **2011**, *43*, 665–73, doi:10.1152/physiolgenomics.00145.2010.

296. McPherson, N.O.; Owens, J.A.; Fullston, T.; Lane, M. Preconception diet or exercise intervention in obese fathers normalizes sperm microRNA profile and metabolic syndrome in female offspring. *Am. J. Physiol. Metab.* **2015**, *308*, E805–E821, doi:10.1152/ajpendo.00013.2015.

297. Silveira, A.C.; Fernandes, T.; Soci, Ú.P.R.; Gomes, J.L.P.; Barretti, D.L.; Mota, G.G.F.; Negrão, C.E.; Oliveira, E.M. Exercise training restores cardiac microRNA-1 and microRNA-29c to nonpathological levels in obese rats. *Oxid. Med. Cell Longev.* **2017**, *2017*, 1–12, doi:10.1155/2017/1549014.

298. Aoi, W.; Ichikawa, H.; Mune, K.; Tanimura, Y.; Mizushima, K.; Naito, Y.; Yoshikawa, T. Muscle-enriched microRNA miR-486 decreases in circulation in response to exercise in young men. *Front. Physiol.* **2013**, *4*, 80, doi:10.3389/fphys.2013.00080.

299. Cui, S.F.; Wang, C.; Yin, X.; Tian, D.; Lu, Q.J.; Zhang, C.Y.; Chen, X.; Ma, J.Z. Similar responses of circulating microRNAs to acute high-intensity interval exercise and vigorous-intensity continuous exercise. *Front. Physiol.* **2016**, *7*, 102, doi:10.3389/fphys.2016.00102.

300. Banzet, S.; Chennaoui, M.; Girard, O.; Racinais, S.; Drogou, C.; Chalabi, H.; Koulmann, N. Changes in circulating microRNAs levels with exercise modality. *J. Appl. Physiol.* **2013**, *115*, 1237–44, doi:10.1152/japplphysiol.00075.2013.

301. Zhang, T.; Birbrair, A.; Wang, Z.M.; Messi, M.L.; Marsh, A.P.; Leng, I.; Nicklas, B.J.; Delbono, O. Improved knee extensor strength with resistance training associates with muscle specific miRNAs in older adults. *Exp. Gerontol.* **2015**, *62*, 7–13, doi:10.1016/j.exger.2014.12.014.

302. Ogasawara, R.; Akimoto, T.; Umeno, T.; Sawada, S.; Hamaoka, T.; Fujita, S. MicroRNA expression profiling in skeletal muscle reveals different regulatory patterns in high and low responders to resistance training. *Physiol. Genomics* **2016**, *48*, 320–324, doi:10.1152/physiolgenomics.00124.2015.

303. Radom-Aizik, S.; Zaldivar, F.; Oliver, S.; Galassetti, P.; Cooper, D.M. Evidence for microRNA involvement in exercise-associated neutrophil gene expression changes. *J. Appl. Physiol.* **2010**, *109*, 252–61, doi:10.1152/japplphysiol.01291.2009.

304. Denham, J.; Prestes, P.R. Muscle-enriched micrornas isolated from whole blood are regulated by exercise and are potential biomarkers of cardiorespiratory fitness. *Front. Genet.* **2016**, *7*, 196, doi:10.3389/fgene.2016.00196.

305. Zhang, T.; Brinkley, T.E.; Liu, K.; Feng, X.; Marsh, A.P.; Kritchevsky, S.; Zhou, X.; Nicklas, B.J. Circulating MiRNAs as biomarkers of gait speed responses to aerobic exercise training in obese older adults. *Aging (Albany. NY).* **2017**, *9*, 900–913, doi:10.18632/aging.101199.
